# Screening and Retaining Adolescents Recruited Through Social Media: Secondary Analysis from a Longitudinal Clinical Trial

**DOI:** 10.2196/47984

**Published:** 2024-02-28

**Authors:** Margaret Weisblum, Emma Trussell, Traci Schwinn, Andrea R Pacheco, Paige Nurkin

**Affiliations:** 1 School of Social Work Columbia University New York, NY United States; 2 Department of Psychology Georgetown University Washington, DC United States; 3 School of Medicine University of Queensland Brisbane Australia; 4 School of Medicine Ochsner Health New Orleans, LA United States; 5 National Institute of Allergy and Infectious Diseases, International Center of Excellence in Research Phnom Penh Cambodia

**Keywords:** adolescents, attrition prevention, Instagram, LGBQ, online recruitment, retention, screening, sexual minority, social media, youth

## Abstract

**Background:**

Social media has become a popular method to recruit participants, particularly for studies with hard-to-reach populations. These studies still face challenges in data quality and, for longitudinal studies, sample retention. However, in addition to aiding in recruitment, social media platforms can help researchers with participant verification and tracking procedures during the study. There is limited previous research describing how longitudinal studies can use social media to screen and retain participants.

**Objective:**

This paper describes strategies implemented to screen and retain a nationwide sample of sexual minority youth who were recruited through social media platforms for a longitudinal study testing a drug abuse prevention program.

**Methods:**

Our screening strategies for participants included collecting necessary demographic information (name, phone, email, and social media accounts), verifying this information using publicly available web-based records, and sending confirmation emails to ensure working email addresses and correct dates of birth. Retention strategies included communications designed to develop positive participant relationships, incentives for survey completion, regular updating of participant contact information, targeting hard-to-reach participants, and using social media as an alternative means of contacting participants.

**Results:**

During enrollment, although the only demographic data required were a phone number and an email address, 87.58% (1065/1216) of participants provided their Instagram as an alternative means of contact. This form of alternative communication remains the most preferred with 87.40% (1047/1198) of participants continuing to provide an Instagram username as of January 2023, about 3 years after recruitment began. In comparison, other alternative means of contact (eg, Facebook and alternative email) were provided by only 6.43% (77/1198) to 56.18% (673/1198) of participants. Direct messaging on Instagram was used to successfully confirm participant identity, remind participants to take annual follow-up surveys, and update lost participant contact information. Screening and retention strategies used in the study have helped achieve 96.30% (1171/1216) to 96.79% (1177/1216) sample retention across 3 waves of data collection.

**Conclusions:**

Though social media can be a helpful tool to recruit participants, attrition and participant authenticity difficulties may be associated with this method. Screening and retention strategies can be implemented to improve retention. Internet searches are effective for screening youth to ensure they meet eligibility requirements. Additionally, social media—Instagram in this study—can help to track and locate participants who do not respond to traditional contact methods.

**Trial Registration:**

ClinicalTrials.gov NCT03954535; https://clinicaltrials.gov/study/NCT03954535

## Introduction

With the rise in social media popularity, web-based recruitment methods for clinical trials have become increasingly popular. Social media allows researchers greater access to nationwide samples [1]; adolescents [2-6]; and hard-to-reach populations [7,8], such as sexual minority individuals [9-13] and people who use substances [14-16]. However, some research has associated web-based recruitment with lower retention rates than in-person recruitment methods [17,18]; researchers have theorized that web-based recruitment lacks the connection and commitment from participants that come from in-person recruitment [18]. Further, there is a greater opportunity in longitudinal studies to lose participants over time due to changes in contact information or the desire to no longer participate in the study [19]. Thus, longitudinal studies that recruit through social media are at high risk for participant attrition.

Despite these challenges, researchers have identified methods to increase retention rates of samples recruited on the web, including frequent communication between surveys [20], financial incentives [21], and building positive rapport with participants [21,22]. Previous research has been able to maintain high retention rates after recruiting participants on social media. One study recruited youth aged between 12 and 25 years using advertisements on social media, Google, Craigslist, and a web-based neighborhood forum; they found retention rates of 78.11% at the 3-month follow-up and 72.18% at the 6-month follow-up [23]. Another study recruited using a similar method of advertising on social media, a collaborating website, and a newsletter and found a retention rate of 88.4% at the 2-week follow-up [24]. Our previous research has used social media (eg, Facebook advertisements) to recruit youth for 2 longitudinal web-based drug abuse prevention programs that maintained retention rates of 97% at the 1-year follow-up [25] and 84.75% at 3-month follow-up [13].

Much of what researchers know about using social media recruitment strategies comes from reports using Facebook. Several studies and systematic reviews have confirmed that advertising on Facebook is more cost-effective and time-efficient than in-person recruitment [7,8,14,15,26,27]. Facebook has also been a valuable tool for locating and communicating with participants in longitudinal studies [28,29]. However, trends in social media have shifted in recent years, especially among younger demographics. In 2015, 71% of teenagers reported using Facebook, while only 52% reported using Instagram [30]. This was notably different in 2022 when 32% of teens reported using Facebook, while 62% reported using Instagram [31]. Instagram has already been used as a successful tool in recruiting sexual and gender minority adolescents and young adults [32-38]. Thus, in 2020, we used Facebook and Instagram to recruit for Free2b, a nationwide 5-year web-based drug abuse intervention program for sexual minority youth (ClinicalTrials.gov NCT03954535).

Though recruiting on social media is cost-effective, timely, and grants access to large and diverse samples, it does not guarantee the authenticity of participants that in-person recruitment allows [39,40]. Social media recruitment requires a thorough screening process to confirm and ensure the legitimacy and eligibility of potential participants. However, thorough screening processes may lead to a more committed sample that can withstand attrition typically seen in longitudinal studies recruited on the web. Throughout the Free2b study, we also used Facebook and Instagram to verify youth’s identities, maintain contact with participants, and locate hard-to-reach participants. To date, little has been published on the use of social media to screen and retain participants in a longitudinal study. This paper describes how thorough screening processes using internet searches and social media, Instagram in particular for sexual minority youth samples, along with a range of retention strategies, help maintain retention in longitudinal clinical trials for youth recruited through social media.

## Methods

### Social Media–Based Recruitment

We used Facebook ads and Instagram promoted posts to recruit participants for a longitudinal trial of a drug abuse prevention program called Free2b. By clicking an ad or post, youth were taken to the study recruitment website. This website contained a brief consent video about study procedures, duration, compensation, and eligibility criteria (English speaking; aged 15 years or 16 years; US resident; access to the internet through computer or tablet; and identifying as lesbian, gay, bisexual, queer, or questioning [LGBQ]). At the conclusion of the video, youth who were still interested in participating could connect to a web-based informed assent quiz. The quiz assessed youth’s knowledge of study aims, procedures, risks, protections, and compensation. Youth who passed the quiz were then allowed to consent to study participation.

Consented youth were asked to provide demographic information: first name, last name, sexual orientation, date of birth, primary and alternative email, primary and alternative phone number, social media handles (Instagram, Facebook, Twitter, and alternative social media), zip code, and alternate contact information (optional). Youth were expressly told that the alternate contact would only be used if their other forms of contact no longer worked. IP addresses were automatically collected upon form submission.

### Eligibility Screening and Enrollment

**Figure 1:**
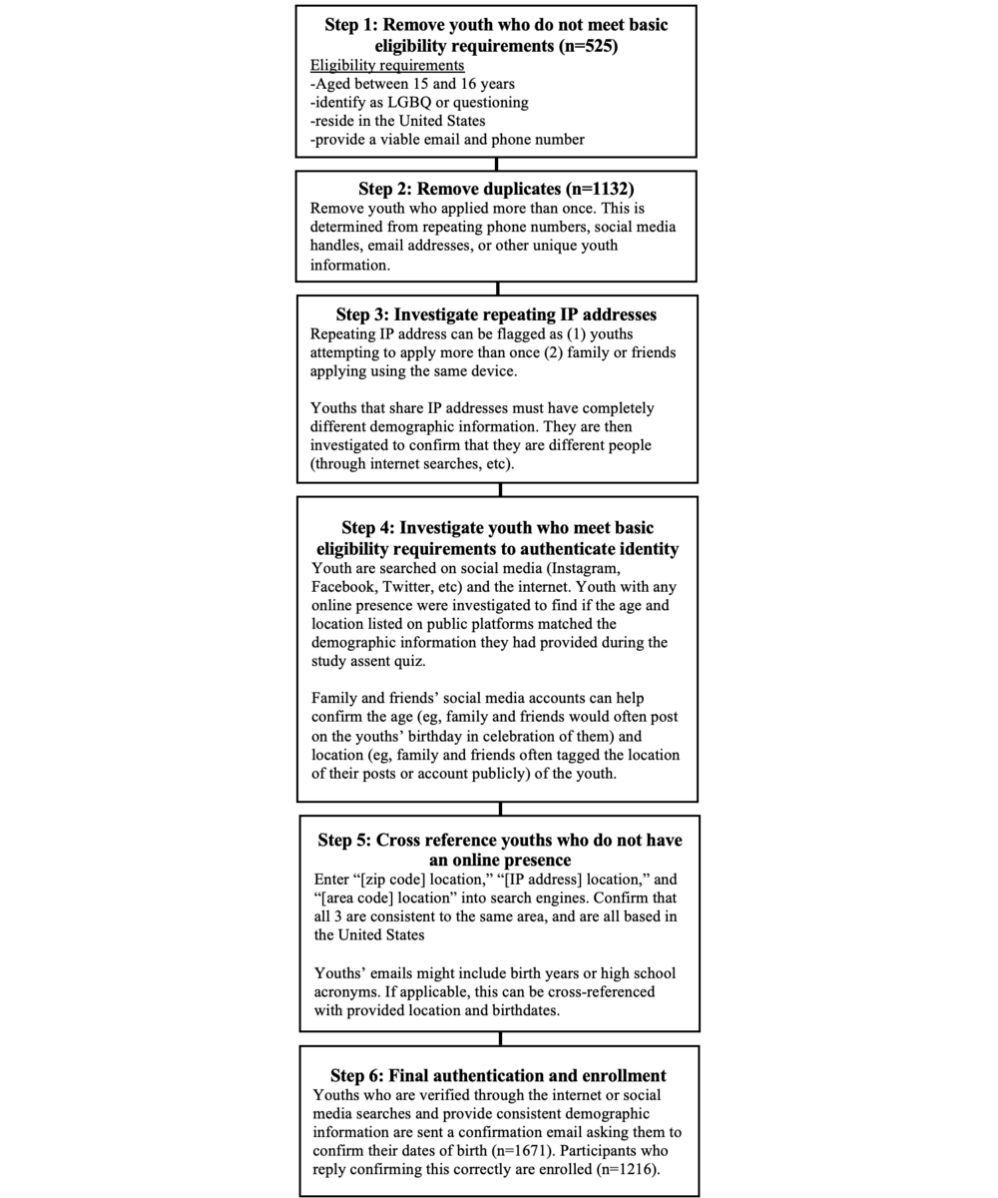
Flowchart of screening strategies implemented during the recruitment phase. LGBQ: lesbian, gay, bisexual, queer, or questioning.

The process to screen consented youth was systemized for research assistants (RAs). RAs were trained to use the steps outlined in Figure 1 to help ensure the authenticity of consenting youth. First, we removed youth who did not meet eligibility requirements: aged between 15 and 16 years, identify as LGBQ or questioning, reside in the United States, and have a phone number and email address. We then removed duplicate names, phone numbers, email addresses, and IP addresses.

To the extent possible, the demographic data from youth who had consented were cross-referenced with information from web-based searches and the social media handles the youth provided. Google searches of names with zip codes often confirmed their existence, location, and age. For example, high school athletes may have profiles showing their names and grades in school. Social media accounts could confirm age and location. Instagram and Twitter bios frequently contained age, high school, and city. Posts and tagged posts were also useful when they referenced birthday celebrations. Confirming sexual orientation was not a required element of screening as many adolescents have not publicly disclosed their sexual orientation and because sexual orientation often changes during adolescence [41]. However, when a lesbian, gay, bisexual, trans, queer, or questioning [LGBTQ] symbol or post was present on youth’s social media, it was noted as a point of authenticity; the lack of such content did not exclude youth from the study. For youth with private or limited social media accounts or no web-based presence, we used other methods to help confirm their identities. For instance, an IP address, cell phone area code, and zip code that correlated helped verify a youth’s authenticity; sometimes an email address included a birth year that matched their provided age or included a name that matched their provided first and last name.

Once youth cleared the aforementioned steps, we sent them an email asking them to reply back confirming the contact information they provided after consenting and we asked them to provide us with their birthdate. Only youth who replied to this email and who accurately confirmed the birthdate they provided during consent were enrolled in the study and randomly assigned to a study condition.

### Building Positive Relationships With Participants

Building rapport with participants is important in longitudinal studies to help maintain retention [42,43]. RAs were trained to use a friendly and appreciative communication style to communicate with participants through phone calls, text messages, emails, and direct messages (DMs) on social media. The language used in messages and calls was positive, supportive, understanding, and appreciative of participants’ time. For example, RAs frequently started messages with language that acknowledged participants’ busy schedules (eg, “I know it’s the beginning of the school year and things are probably pretty hectic right now.”) to convey an understanding that the study surveys were unlikely to be their priority.

Given our understanding that participants were busy, we maintained the philosophy that no participant is “lost” unless we have no working contact information. However, even when a participant met the standards to be considered “lost,” they were not removed from the study. This allowed us to recover participants who may have chosen to skip a survey one year, but then chose to take the next year’s survey.

**Figure 2:**
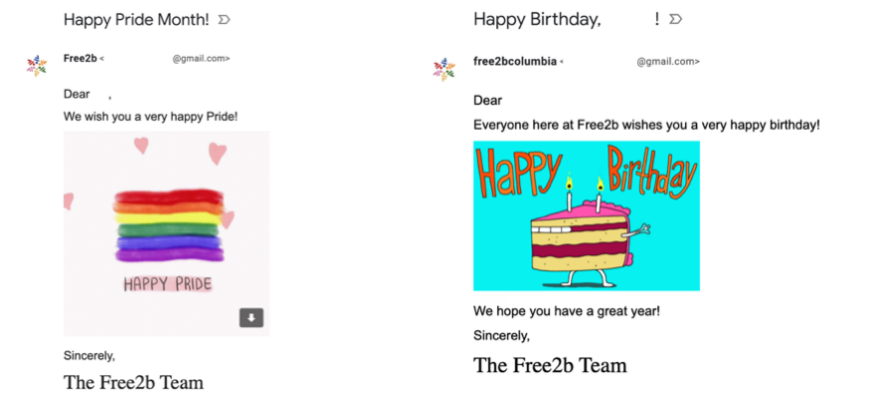
Examples of holiday and birthday messages sent to participants throughout the study.

We also built positive relationships by honoring when participants requested needing more time to complete a survey (eg, during finals week). Annual holiday and birthday texts and emails (Figure 2) helped maintain contact but also served to build rapport. Email correspondence encouraged participants to contact us with questions or concerns and included the study phone number and the principal investigator’s phone number and email to facilitate this contact.

### Communication Strategies

Given the importance of sample retention to longitudinal research, timely communication with participants is essential. Project email and social media accounts were checked regularly; RAs were expected to respond immediately to participants’ texts, phone calls, emails, or DMs on social media. Each interaction was logged in a shared database to record contact history. This record helped determine the best methods of contact for a participant. Before making contact, RAs read through a participant’s contact log for previous successful contacts (eg, a participant might respond to texts more often than calls). RAs were also instructed to vary their contact methods, switching between text, email, voicemail, or social media. These methods increased the chances of participants seeing our communication attempts. We also made an effort to send messages with different wording or images (eg, for holiday cards each year) to participants, rather than repeatedly sending the same template message. This helped our communication come across as individually tailored, rather than as an automated message to all participants.

Finally, RAs were instructed to maintain frequent communication and reminders without overwhelming participants. As described earlier, they often started texts and phone calls with understanding language. Additionally, most reminders to take surveys included a link so participants would not have to go through their inbox to find the original survey reminder. When talking to participants on the phone, RAs always offered to send a follow-up text or email with the survey link. Finally, if a participant had not taken a survey after numerous reminders, or they mentioned that they are busy with other activities, we offered to pause communications and asked them when they would like us to check back.

### Update Contact Info Surveys

To minimize the likelihood of losing participants between annual surveys due to changes in their contact information, we attempted to update participant contact information quarterly. We provided participants with their phone number or phone numbers; email address or email addresses; social media account handles; zip code; and if they have provided one, their alternate contact’s phone number in a brief web-based form. If all of their contact information was up to date, they simply clicked “correct,” or they could update their information if necessary. Youth could also continue to provide no alternate contact, add an alternate contact, remove the alternate contact they had provided, or change the one they provided.

**Figure 3:**
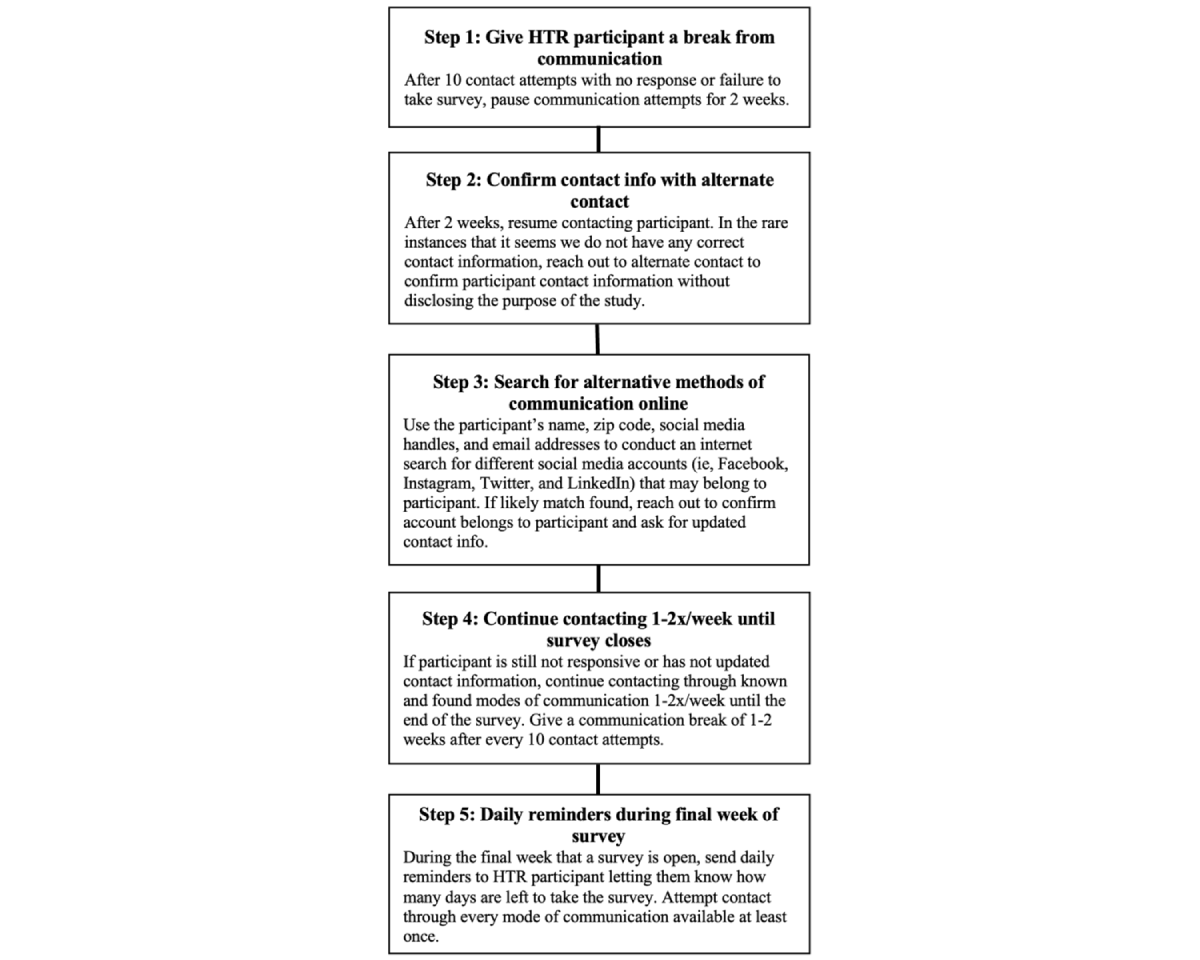
Flowchart of steps taken to contact hard-to-reach (HTR) participants and remind them to complete annual surveys.

Inevitably, some participants are lost over time due to frequent changes in contact information, competing priorities for time, or loss of interest in continuing in the study [44]. If we reached out to a participant more than 10 times with no response, they were considered “hard-to-reach.” RAs were trained on standard protocols for contacting hard-to-reach participants (Figure 3). Once a participant became “hard-to-reach,” we took a break from contacting them for several weeks. We then conducted an internet search and used social media to try to reconnect. In the rare instance that a cell phone and email was no longer working, and we were confident we were no longer reaching the participant, we discreetly reached out to their alternate contact without disclosing the purpose of the study. Youth who were considered “hard-to-reach” were always asked if they would like to be removed from the study or take a break from participation so that we did not bother them unnecessarily or needlessly spend time trying to collect survey data. Though some youth who did not respond to our survey reminders may no longer have wished to participate, we did not make this decision for them.

### Ethical Considerations

Study procedures were approved by the Columbia University Institutional Review Board (IRB-AAAR5072). A waiver of parental permission was granted to reduce risks; such waivers may also increase participation from adolescents who are not out to their parents [45]. Because our sample could be considered a vulnerable population, a detailed data and safety and monitoring plan was also established, and a Data and Safety Monitoring Board met no less than once a year. A primary charge for the Data and Safety Monitoring Board in year 1 was to review Institutional Review Board–approved recruitment and informed consent procedures.

After completing the aforementioned consent processes, participants received US $30, US $35, US $40, US $45, and US $50 for each of the 5 waves of data collection (pretest; posttest; and 1-, 2-, and 3-year follow-up, respectively). Participants were able to choose from several e-gift card options. As soon as a survey was completed, participants were notified that they would receive their e-gift card within 36 hours. Sending e-gift cards in a timely manner showed our appreciation and helped maintain positive relationships with participants. We also reminded participants to redeem their gift cards when they were close to expiring.

## Results

### Rates of Provided Participant Contact Information

All participants were required to provide a primary email and phone number in order to be enrolled in the study, but 11.76% (143/1216) also provided an alternative phone number and 51.23% (623/1216) provided an alternative email. At the time of recruitment, 87.58% (1065/1216) of enrolled Free2b participants provided an Instagram account as part of their contact information, as compared to 19.98% (243/1216) providing an alternate contact (eg, family or friends), 15.13% (184/1216) providing a Facebook account, and 29.03% (353/1216) providing an alternative social media account (eg, Twitter, Tumblr, or TikTok). Only 10.61% (129/1216) provided no form of alternate contact or social media accounts (Table 1). The percentage of participants with an Instagram account has remained relatively stable in the approximately 3 years since recruitment. Throughout the study, Instagram has remained the most commonly provided alternative method of contact. As of January 2023, a total of 87.40% (1047/1198) of Free2b participants have provided Instagram handles, while only 22.37% (268/1198) of participants have provided an alternate contact with a cell phone number, 19.37% (232/1198) have provided a Facebook account, 30.47% (365/1198) have provided an alternative social media account, 6.43% (77/1198) have provided an alternative phone number, and 56.18% (673/1198) have provided an alternative email.

**Table 1:** Number of participants who provided each type of alternative contact information at enrollment and 3 years after recruitment.

Type of contact information	At enrollment (2020; n=1216), n (%)	Currently (January 2023; n=1198), n (%)
Alternate contact	243 (19.98)	268 (22.37)
Alternative email	623 (51.23)	673 (56.18)
Alternative number	143 (11.76)	77 (6.43)
Facebook	184 (15.13)	232 (19.37)
Instagram	1065 (87.58)	1047 (87.40)
No alternate contact number or social media	129 (10.61)	117 (9.77)
Other social media	353 (29.03)	365 (30.47)

### Direct Messaging and Locating Hard-to-Reach Participants Through Social Media

If participants have not taken their surveys after multiple automated reminders, they are added to a “call list” to receive personalized communication from RAs. First, RAs attempt to contact them through traditional contact methods (phone calls, text messages, and emails); if this is not effective, they begin adding social media contacts (eg, direct messaging on Instagram) in addition to traditional methods. Of the 17 participants on the call list for Survey 1 who completed the survey, 100% received only traditional contacts, and 0% received a combination of traditional and social media contacts. Of the 102 participants on the call list for Survey 2 who completed the survey, 71.6% (73/102) received only traditional contacts and 28.4% (29/102) received a combination of traditional and social media contacts. Of the 100 participants on the call list for Survey 3 who completed the survey, 77% (77/100) received only traditional contacts and 23% (23/100) received a combination of traditional and social media contacts. Finally, of the 121 participants on the call list during Survey 4 who completed the survey, 81.8% (99/121) received only traditional contacts and 18.2% (22/121) received a combination of traditional and social media contacts (Table 2).

**Table 2:** The percentage of participants who were on the call list and then took the survey after traditional contact (phone and email) versus a combination of traditional and social media (eg, Instagram direct messages) contacts.

Survey number	Combination of traditional and social media contacts, n (%)	Traditional contacts (phone and email), n (%)
Survey 1 (n=17)	0 (0)	17 (100)
Survey 2 (n=102)	29 (28.4)	73 (71.6)
Survey 3 (n=100)	23 (23)	77 (77)
Survey 4 (n=121)	22 (18.2)	99 (81.8)

### Social Media Versus Traditional Contact Methods for Survey Reminders

**Figure 4:**
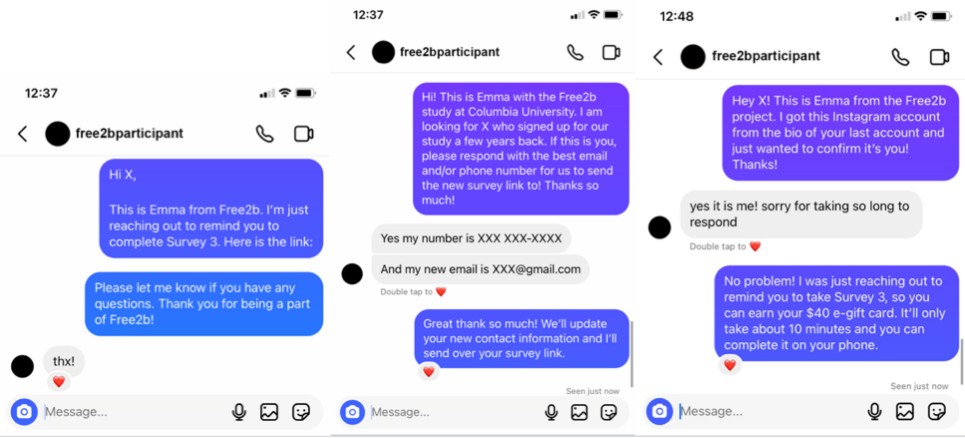
Examples of interactions with participants through direct messages on Instagram that have been recreated and anonymized.

Both Instagram and Facebook offer a direct messaging feature. However, unlike our attempts to communicate with participants through Facebook DMs, communication through Instagram DMs was frequently successful. As seen in the left screenshot in Figure 4, an anonymized recreation of an interaction with a study participant, Instagram DMs resulted in direct replies or liked messages. Instagram also notified us when the participants read our DMs by displaying “seen” under read messages. Moreover, success of Instagram survey reminders was evidenced by the completion of surveys soon after DM reminders: of the participants who received social media contacts, 41% (12/29) completed Survey 2, 39% (9/23) completed Survey 3, and 27% (6/22) completed Survey 4 within 48 hours of being sent a reminder through DM. In particular, Instagram proved to be a useful method for contacting hard-to-reach participants, who otherwise did not respond to calls, texts, and emails to take follow-up surveys. Attempts to contact participants on Facebook did not yield similar results.

Instagram was also a helpful tool for finding lost participants for whom we had no working contact information. When a participant was “lost,” we used Google to search for the participant’s new social media accounts. Both Facebook and Instagram were used to DM lost participants, but only Instagram resulted in successful participant discoveries (middle and right screenshots in Figure 4). Despite the success that these examples indicate, there were still instances where our Instagram DMs were ignored, never seen, or we were simply unable to send messages to participant accounts.

### Retention Strategies

Our incentives increased in value as the study continued and ranged from US $30-US $50. These incentives are almost always claimed by the participants: 99.59% (1208/1213) claimed their Survey 1 gift card, 99.41% (1170/1177) claimed their Survey 2 gift card, and 99.32% (1168/1176) claimed their Survey 3 gift card. Of the few participants who did not claim their gift cards, only 1 or 2 participants per survey explicitly stated that they did not want their gift card.

Our success in developing and maintaining positive relationships with participants has been demonstrated by the messages we occasionally receive from participants expressing their appreciation for the project:

Example 1:

Hi Free2b folks, I just wanted to send a message that the project has seen me through a lot of change (both with time and otherwise)...Thank you for providing this space.

Example 2:

I appreciate you 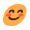 . I am glad to be apart [sic] of this after so many years (it’s very exciting).

Example 3:

Thank you! I’m not sure if this is a no-reply kind of thing, but in the case that it’s not, I want to show my gratitude! Thank you for the opportunity to be in this study, it really means a lot to me. I am very excited to see the impact this will have! And the money has helped me so much, I was able to pay for my first binder with it! I am eternally grateful.

Example 4:

Good morning to whoever is reading this!!! Hi I’m ______ and I’m a part of the Free2b program and I want to say thank you! Your website has really allowed me to open up with those around me, as a bisexual and proud young female.

Such messages from participants frequently express gratitude, that the study is helpful for them, interest in the outcome of the research, or that the project has had a positive impact on how they feel about their sexual orientation.

Finally, 3 Update Contact Info Surveys were sent in between each study survey. Participants were not incentivized to complete these surveys, but still, 87.14% (1057/1213) of participants completed at least 1 Update Contact Info Survey between Survey 2 and 3 and 84.17% (1021/1213) completed at least 1 between Survey 3 and Survey 4.

### Retention Rates

Our Instagram and Facebook recruitment and screening techniques allowed us to verify and enroll 1216 participants in the Free2b study. Retention at the first survey after enrollment was 99.75% (1213/1216). The second survey, taken about 4 months later, was completed by 96.79% (1177/1216) of participants. We were able to maintain similarly high retention at 96.71% (1176/1216) in the 1-year follow-up survey (Survey 3) and 96.30% (1171/1216) in our 2-year follow-up (Survey 4). Of the participants who did not take Survey 2, a total of 28% (10/36) were recovered in Survey 3. Of the participants who took Survey 2 but not Survey 3, a total of 9% (1/11) were recovered in Survey 4. Additionally, of the participants who did not take Surveys 2 or 3, a total of 17% (5/30) were recovered in Survey 4. Overall, since the end of the recruitment period, we have only lost 6 participants (<1%), as defined by a participant not completing any follow-up surveys to date and having zero working contact information. Despite the difficulty in reaching these participants, they are still invited to participate in each survey, and we contact them each year.

The 3 participants who did not take Survey 1 were exited from the study, and 15 participants asked to exit the study after the first survey. After Survey 2, one additional participant asked to be exited, bringing our total exited participants to 19.

## Discussion

### Principal Findings

Due to the increased popularity of social media and other messaging platforms, researchers have more opportunities to identify and communicate with participants in longitudinal clinical trials. This paper reports on the use of social media to aid participant screening and retention in a longitudinal study for sexual minority youth. Our findings suggest that a sample recruited through social media platforms can achieve minimal attrition with certain screening and retention strategies. Our results confirm the effectiveness of commonly published retention strategies [42,43,46] and offer new methods, such as using social media to maintain contact with participants. Study findings further suggest that Instagram is an effective method for communicating with and finding potentially lost participants over Facebook for sexual minority youth. This points to the importance of researchers following social media trends to meet their potential or enrolled participants where they are.

Overall, the various retention and screening strategies used in this study have shown promising results, with retention rates ranging from 96.30% (1171/1216) to 96.79% (1177/1216) in follow-up surveys. Our retention rates are somewhat higher than other studies with sexual minority youth. In a study of sexual minority youth whose sample included minors, retention rates at each study wave ranged from 82% to 90% [47]. Another study of sexual minority youth aged between 18 and 19 years found retention rates ranging from 85.9% to 89.5% [48].

Sexual minority youth are a hard-to-reach population, making it difficult to recruit using in-person methods [49]. However, social media has presented an accessible way to reach this demographic [1,2,5,6]. We used Instagram and Facebook to recruit a large nationwide sample of sexual minority youth. But studies that use web-based recruitment can be vulnerable to poor data quality as eligibility is harder to verify compared to in-person methods [40]. A common strategy in web-based recruitment is the use of eligibility screening questions to remove ineligible applicants [39,50]. However, screening questions do not guarantee authentic answers. Moreover, when studies such as ours compensate participants, duplicate or fraudulent enrollees are common [51]. Therefore, the benefits of web-based recruitment can be offset by risks to sample validity and data integrity. There is limited literature outlining the extent of these threats and how to mitigate them [40].

By cross-referencing demographic data provided by the participant with publicly available information and confirming participant birthdates through email, we improved the overall quality of this study data. If we had not validated their email by asking for their birthday confirmation, we may have enrolled people in the study who provided inactive email addresses and who were not 15-16 years old. Without collecting sufficient contact information at the study’s onset—primary and alternative phone numbers, primary and alternative emails, social media handles, and an optional alternate contact—sample retention would likely have been lower.

As seen in Table 1, Instagram was consistently the most common alternative contact method youth provided. This is unsurprising given the popularity of Instagram among our age demographic [31]. This may also be due to our recruitment methods through Instagram. Many participants had already interacted with us on Instagram—through DMs, comments, and likes—and thus may have been more comfortable sharing their handle. We have continued to use Instagram to reach participants throughout the duration of the study given its continued popularity among the sample. During survey data collection, reminders through Instagram DM were successful, as seen through the participants who took the survey within 48 hours of receiving a reminder DM. Instagram DMs were also useful to help update participant contact information. Understanding the most popular form of social media among a recruited demographic may help researchers to remain in contact with hard-to-reach participants.

The retention strategies used in this study include those traditionally used in longitudinal clinical trials as well as new methods that reflect the current shifts in social media trends. Traditional methods include survey incentives, building positive relationships with participants, and regularly updating participant contact information [42,43,52]. The incentives for each survey were popular as evidenced by the high number of gift card acceptances after each survey. Training RAs to have consistent communication standards and demonstrate respect for participants’ time helped us build rapport. Evidence of our success at building these positive relationships includes when they frequently thanked us for their birthday or holiday messages or upon receipt of their gift card.

Worth noting are the examples of participant messages outlined in our results. Sometimes participants were unsure if they were emailing a “real person” (eg, “not sure if this is a no-reply kind of thing”). This concern likely resulted from the use of templates for mass emails related to surveys or gift cards. Though we personalized these emails with first names, the concerns voiced by some participants is an important reminder that adolescents are savvy and able to detect when correspondence is mass generated versus individually written. Researchers may benefit from ensuring they have a mix of automated and individualized messages, as we did, to maintain positive relationships.

Throughout the study we reached out to participants to update their contact information through a brief survey. This task required minimal effort on behalf of the participant. The ease of use of the survey likely contributed to the high rates of completion. In turn, the correct contact information minimized attrition. These surveys may have also helped us to maintain positive relationships with our participants as we were able to note changes in names, pronouns, and gender identities, thereby minimizing the chance to use a deadname or misgender a participant, which can be detrimental when maintaining rapport with sexual minority youth [53].

Tracking lost participants and finding alternative methods to contact hard-to-reach participants are both crucial to prevent attrition. Throughout the study, we used Instagram to reach out to hard-to-reach participants as an alternative contact method when calls, texts, and emails were ineffective. Social media contacts were successful as seen when participants took their survey within 48 hours of receiving a DM reminder from us. This success is likely attributable to participants’ frequent use of the app; those who were active on the platform may have been more likely to see our DMs over calls, texts or emails which can be deemed spam.

When tracking lost participants, we implemented multiple strategies. Researchers have commonly used multiple forms of web-based methods to track participants: search engines (eg, Google) and fee-based directories (eg, White Pages) are 2 common examples [54]. Though search engines are useful to locate participants, they often do not provide new methods of contact. Therefore, we used social media to locate and DM potentially lost participants. Social media platforms, primarily Facebook, have also been used by researchers to search for participants. Despite the reported success shown on tracking through Facebook [28,54,55], we have primarily used Instagram over Facebook due to its higher popularity among our sample (Table 1).

Unless requested to be exited, no participant was considered lost from the study. We used social media to “recover” participants whom we lost contact with due to changes in their contact information. After finding a profile on Instagram that matched their demographic information, we reached out to participants regarding their participation in the study (middle screenshot in Figure 4). In some instances, we were also able to use old accounts to update contact information. After attempting to reach some participants through Instagram, we found that their accounts were no longer active, but they had added a link to their new account in their bio through which we were able to reach them (right screenshot in Figure 4). Overall, Instagram has been useful as an alternative contact method for survey reminders, to track down lost participants, and to build positive relationships with participants.

### Limitations

A limitation of using Instagram to screen and maintain contact with participants is that Instagram frequently changes its policies, including how DMs can be sent. In March 2021, Instagram announced it would be banning adults from direct messaging teenagers under the age of 18 years who do not follow the adult’s account [56]. This may affect retention efforts when using Instagram as a contact method in a sample of youth. It is unclear how this policy will change in the future. Moreover, people can easily change their profile handle names, preventing us from finding previously provided accounts. An additional limitation of using social media as a method to recover participants is that these methods are more effective with participants who have uncommon names, as it was very difficult to find participants on social media if there were hundreds or thousands of users with the same name. Finally, efforts to communicate or contact participants through social media were likely less effective for participants who were not out or did not want to be publicly associated with our Instagram account.

### Conclusion

This paper demonstrates effective screening and retention methods to conduct a longitudinal clinical trial for sexual minority youth. Social media, particularly Instagram, was found to be useful both in the screening process and in maintaining contact with participants throughout the study. Through the use of similar thoughtful screening and retention strategies, others may be able to replicate our high retention rates. Future research is needed to determine the efficacy of individual strategies, as well as to test these strategies in different populations and on new social media platforms as they gain popularity.

## Abbreviations

DM: direct message
LGBTQ: lesbian, gay, bisexual, trans, queer, or questioning
LGBQ: lesbian, gay, bisexual, queer, or questioning
RA: research assistant

## References

[1] Schrager SM, Goldbach JT, Mamey MR (2018). Development of the sexual minority adolescent stress inventory. Front Psychol.

[2] Altshuler AL, Storey HLG, Prager SW (2015). Exploring abortion attitudes of US adolescents and young adults using social media. Contraception.

[3] Chu JL, Snider CE (2013). Use of a social networking web site for recruiting Canadian youth for medical research. J Adolesc Health.

[4] Ellis LA, Collin P, Davenport TA, Hurley PJ, Burns JM, Hickie IB (2012). Young men, mental health, and technology: implications for service design and delivery in the digital age. J Med Internet Res.

[5] Fenner Y, Garland SM, Moore EE, Jayasinghe Y, Fletcher A, Tabrizi SN, Gunasekaran B, Wark JD (2012). Web-based recruiting for health research using a social networking site: an exploratory study. J Med Internet Res.

[6] Mustanski B, Greene GJ, Ryan D, Whitton SW (2015). Feasibility, acceptability, and initial efficacy of an online sexual health promotion program for LGBT youth: the queer sex ed intervention. J Sex Res.

[7] Thornton L, Batterham PJ, Fassnacht DB, Kay-Lambkin F, Calear AL, Hunt S (2016). Recruiting for health, medical or psychosocial research using Facebook: systematic review. Internet Interv.

[8] Whitaker C, Stevelink S, Fear N (2017). The use of Facebook in recruiting participants for health research purposes: a systematic review. J Med Internet Res.

[9] Hernandez-Romieu AC, Sullivan PS, Sanchez TH, Kelley CF, Peterson JL, Del Rio C, Salazar LF, Frew PM, Rosenberg ES (2014). The comparability of men who have sex with men recruited from venue-time-space sampling and facebook: a cohort study. JMIR Res Protoc.

[10] Buckingham L, Becher J, Voytek CD, Fiore D, Dunbar D, Davis-Vogel A, Metzger DS, Frank I (2017). Going social: success in online recruitment of men who have sex with men for prevention HIV vaccine research. Vaccine.

[11] Vial AC, Starks TJ, Parsons JT (2014). Finding and recruiting the highest risk HIV-negative men who have sex with men. AIDS Educ Prev.

[12] Parsons JT, Vial AC, Starks TJ, Golub SA (2013). Recruiting drug using men who have sex with men in behavioral intervention trials: a comparison of internet and field-based strategies. AIDS Behav.

[13] Schwinn TM, Thom B, Schinke SP, Hopkins J (2015). Preventing drug use among sexual-minority youths: findings from a tailored, web-based intervention. J Adolesc Health.

[14] Ramo DE, Prochaska JJ (2012). Broad reach and targeted recruitment using Facebook for an online survey of young adult substance use. J Med Internet Res.

[15] Ramo DE, Rodriguez TMS, Chavez K, Sommer MJ, Prochaska JJ (2014). Facebook recruitment of young adult smokers for a cessation trial: methods, metrics, and lessons learned. Internet Interv.

[16] Craig SL, McInroy LB, D'Souza SA, Austin A, McCready LT, Eaton AD, Shade LR, Wagaman MA (2017). Influence of information and communication technologies on the resilience and coping of sexual and gender minority youth in the United States and Canada (project #queery): mixed methods survey. JMIR Res Protoc.

[17] Bajardi P, Paolotti D, Vespignani A, Eames K, Funk S, Edmunds WJ, Turbelin C, Debin M, Colizza V, Smallenburg R, Koppeschaar C, Franco AO, Faustino V, Carnahan A, Rehn M, Merletti F, Douwes J, Firestone R, Richiardi L (2014). Association between recruitment methods and attrition in Internet-based studies. PLoS One.

[18] Lane TS, Armin J, Gordon JS (2015). Online recruitment methods for web-based and mobile health studies: a review of the literature. J Med Internet Res.

[19] Hill KG, Woodward D, Woelfel T, Hawkins JD, Green S (2016). Planning for long-term follow-up: strategies learned from longitudinal studies. Prev Sci.

[20] Nelson KM, Ramirez JJ, Carey MP (2017). Developing online recruitment and retention methods for HIV prevention research among adolescent males who are interested in sex with males: interviews with adolescent males. J Med Internet Res.

[21] Jong ST, Stevenson R, Winpenny EM, Corder K, van Sluijs EMF (2023). Recruitment and retention into longitudinal health research from an adolescent perspective: a qualitative study. BMC Med Res Methodol.

[22] Temple EC, Brown RF (2011). A comparison of Internet-based participant recruitment methods: engaging the hidden population of cannabis users in research. J Res Pract.

[23] Villanti AC, Vallencourt CP, West JC, Peasley-Miklus C, LePine SE, McCluskey C, Klemperer E, Priest JS, Logan A, Patton B, Erickson N, Hicks J, Horton K, Livingston S, Roemhildt M, Singer E, Trutor M, Williams R (2020). Recruiting and retaining youth and young adults in the Policy and Communication Evaluation (PACE) vermont study: randomized controlled trial of participant compensation. J Med Internet Res.

[24] Wisk LE, Nelson EB, Magane KM, Weitzman ER (2019). Clinical trial recruitment and retention of college students with type 1 diabetes via social media: an implementation case study. J Diabetes Sci Technol.

[25] Schwinn TM, Schinke SP, Hopkins J, Keller B, Liu X (2018). An online drug abuse prevention program for adolescent girls: posttest and 1-year outcomes. J Youth Adolesc.

[26] Loxton D, Powers J, Anderson AE, Townsend N, Harris ML, Tuckerman R, Pease S, Mishra G, Byles J (2015). Online and offline recruitment of young women for a longitudinal health survey: findings from the Australian longitudinal study on women's health 1989-95 cohort. J Med Internet Res.

[27] Sanchez C, Grzenda A, Varias A, Widge AS, Carpenter LL, McDonald WM, Nemeroff CB, Kalin NH, Martin G, Tohen M, Filippou-Frye M, Ramsey D, Linos E, Mangurian C, Rodriguez CI (2020). Social media recruitment for mental health research: a systematic review. Compr Psychiatry.

[28] Mychasiuk R, Benzies K (2012). Facebook: an effective tool for participant retention in longitudinal research. Child Care Health Dev.

[29] Ryan GS (2013). Online social networks for patient involvement and recruitment in clinical research. Nurse Res.

[30] Lenhart A (2015). Pew Research Center.

[31] Vogels EA, Gelles-Watnick R, Massarat N (2022). Teens, social media and technology 2022. Pew Research Center.

[32] Parker JN, Hunter AS, Bauermeister JA, Bonar EE, Carrico A, Stephenson R (2021). Comparing social media and in-person recruitment: lessons learned from recruiting substance-using, sexual and gender minority adolescents and young adults for a randomized control trial. JMIR Public Health Surveill.

[33] Guillory J, Wiant KF, Farrelly M, Fiacco L, Alam I, Hoffman L, Crankshaw E, Delahanty J, Alexander TN (2018). Recruiting hard-to-reach populations for survey research: using Facebook and Instagram advertisements and in-person intercept in LGBT bars and nightclubs to recruit LGBT young adults. J Med Internet Res.

[34] Salk RH, Thoma BC, Choukas-Bradley S (2020). The gender minority youth study: overview of methods and social media recruitment of a nationwide sample of U.S. cisgender and transgender adolescents. Arch Sex Behav.

[35] Zlotorzynska M, Bauermeister JA, Golinkoff JM, Lin W, Sanchez TH, Hightow-Weidman L (2021). Online recruitment of youth for mHealth studies. Mhealth.

[36] Stern MJ, Fordyce E, Hansen C, Viox MH, Michaels S, Schlissel A, Avripas S, Harper C, Johns M, Dunville R (2020). Social media recruitment for a web survey of sexual and gender minority youth: an evaluation of methods used and resulting sample diversity. LGBT Health.

[37] Ybarra M, Goodenow C, Rosario M, Saewyc E, Prescott T (2021). An mHealth intervention for pregnancy prevention for LGB Teens: an RCT. Pediatrics.

[38] Nelson KM, Pantalone DW, Carey MP (2019). Sexual health education for adolescent males who are interested in sex with males: an investigation of experiences, preferences, and needs. J Adolesc Health.

[39] Chandler JJ, Paolacci G (2017). Lie for a dime: when most prescreening responses are honest but most study participants are impostors. Soc Psychol Personal Sci.

[40] Pozzar R, Hammer MJ, Underhill-Blazey M, Wright AA, Tulsky JA, Hong F, Gundersen DA, Berry DL (2020). Threats of bots and other bad actors to data quality following research participant recruitment through social media: cross-sectional questionnaire. J Med Internet Res.

[41] Stewart JL, Spivey LA, Widman L, Choukas-Bradley S, Prinstein MJ (2019). Developmental patterns of sexual identity, romantic attraction, and sexual behavior among adolescents over three years. J Adolesc.

[42] Adamson L, Chojenta C (2014). Developing relationships and retaining participants in a longitudinal study. Int J Mult Res Approaches.

[43] Hanna KM, Scott LL, Schmidt KK (2014). Retention strategies in longitudinal studies with emerging adults. Clin Nurse Spec.

[44] Lyons KS, Carter JH, Carter EH, Rush KN, Stewart BJ, Archbold PG (2004). Locating and retaining research participants for follow-up studies. Res Nurs Health.

[45] Mustanski B (2011). Ethical and regulatory issues with conducting sexuality research with LGBT adolescents: a call to action for a scientifically informed approach. Arch Sex Behav.

[46] Grape A, Rhee H, Wicks M, Tumiel-Berhalter L, Sloand E (2018). Recruitment and retention strategies for an urban adolescent study: lessons learned from a multi-center study of community-based asthma self-management intervention for adolescents. J Adolesc.

[47] Whitton SW, Newcomb ME, Messinger AM, Byck G, Mustanski B (2019). A longitudinal study of IPV victimization among sexual minority youth. J Interpers Violence.

[48] Kapadia F, Bub K, Barton S, Stults CB, Halkitis PN (2015). Longitudinal trends in sexual behaviors without a condom among sexual minority youth: the P18 cohort study. AIDS Behav.

[49] Lucassen MFG, Fleming TM, Merry SN (2017). Tips for research recruitment: the views of sexual minority youth. J LGBT Youth.

[50] Ali SH, Foreman J, Capasso A, Jones AM, Tozan Y, DiClemente RJ (2020). Social media as a recruitment platform for a nationwide online survey of COVID-19 knowledge, beliefs, and practices in the United States: methodology and feasibility analysis. BMC Med Res Methodol.

[51] Lawlor J, Thomas C, Guhin AT, Kenyon K, Lerner MD, Drahota A (2021). Suspicious and fraudulent online survey participation: introducing the REAL framework. Methodol Innov.

[52] Hall AR, Nishina A (2018). Daily compensation can improve college students’ participation and retention rates in daily report studies. Emerg Adulthood.

[53] Sinclair-Palm J, Chokly K (2022). ‘It’s a giant faux pas’: exploring young trans people’s beliefs about deadnaming and the term deadname. J LGBT Youth.

[54] Calderwood L, Brown M, Gilbert E, Wong E, Lynn P (2021). Innovations in participant engagement and tracking in longitudinal surveys. Advances in Longitudinal Survey Methodology. 1st Edition.

[55] Stephenson NL, Hetherington E, Dodd S, Mathews A, Tough S (2019). Mitigation of participant loss to follow-up using Facebook: all our families longitudinal pregnancy cohort. J Med Internet Res.

[56] Vincent J (2021). Instagram will no longer let adults message teens who don’t follow them. The Verge.

